# Molecular Background and Clinical Implications of Glucose Disorders in Patients with Psoriatic Arthritis

**DOI:** 10.3390/jcm12185814

**Published:** 2023-09-07

**Authors:** Bogna Grygiel-Górniak, Weronika Skoczek

**Affiliations:** Department of Rheumatology, Rehabilitation and Internal Diseases, Poznan University of Medical Sciences, 61-701 Poznan, Poland

**Keywords:** psoriatic arthritis, glucose disorders, diabetes type 2, treatment options

## Abstract

Psoriatic arthritis (PsA) is an inflammatory musculoskeletal disease characterized by joint and entheses involvement. This condition is often associated with an increased prevalence of obesity, encompassing more than one-third of all patients. Given the presence of metabolic disorders, it becomes crucial to enhance clinical oversight of metabolic parameters. An early diagnosis of glucose irregularities in PsA allows for the assessment of an effective treatment strategy. The approach proves valuable in preventing the development of insulin resistance (IR) or diabetes mellitus type 2 (DMt2). Similar pathways characterize the pathomechanism of PsA and DMt2, offering an innovative perspective on treatment management. The cytokines and adipokines synthesized in the course of PsA significantly impact the development process of IR and DMt2 in different mechanisms of action. Conversely, glucose disorders influence the activity of PsA and therapy outcomes. Given the chronic inflammatory background shared by PsA, obesity, and DMt2, it is evident that inadequate management of any of the mentioned conditions can exacerbate the others. Thus, when PsA coincides with DMt2, a comprehensive multidimensional approach is necessary. This includes an effective immunosuppressive regimen complemented by appropriate anti-diabetic and insulin therapies. Moreover, often overlooked recommendations concerning overall well-being and lifestyle adjustments hold significance. This manuscript explores the connections and the relationship between the molecular background of PsA and glucose disorders. It provides a detailed exposition of specific therapeutic approaches for both conditions.

## 1. Introduction

Psoriatic arthritis (PsA) is a heterogeneous disease characterized by joint involvement and active or latent psoriasis. PsA typically presents as peripheral or axial polyarthritis with chronic joint damage, potentially leading to disability [1]. Due to the complex mechanism and stimulation of various metabolic pathways in PsA, it is often accompanied by numerous comorbidities. Among the most common are metabolic syndrome, osteoporosis, fatty liver disease, hypertension, ophthalmic disorders, depression, and type 2 diabetes mellitus (DMt2) [2,3].

Metabolic disorders in PsA arise from various pathomechanisms, one of which involves a low-grade inflammation stimulated by adipocytokines’ overproduction [4]. During PsA, inflammation modulates insulin sensitivity and disrupts insulin signaling pathways. Released cytokines downregulate the activity of phosphoinositide-3-kinase (PI-3 kinase), impairing the docking of glucose transporter type 4 (GLUT-4), causing serine autophosphorylation in insulin receptor substrate-1 (IRS-1), increasing the activity of c-Jun NH(2)-terminal kinase (JNK), and interrupting insulin-glucose homeostasis [5,6,7].

Given that the prevalence of obesity in PsA is 38%, the relationship between adipokines, inflammation, and insulin resistance becomes critical in understanding the pathogenesis of glucose disorders in this patient population [5,6]. Thus, managing and controlling DMt2 in patients with PsA poses a significant medical challenge. A comprehensive understanding of the pathomechanism can optimize therapeutic outcomes and significantly improve the quality of life for these patients. This manuscript discusses and explains the role of specific molecular pathways shared between PsA and DMt2. Understanding these pathways can enhance the effectiveness of PsA treatment while simultaneously addressing glucose disorders. Moreover, we explore the implications of specific medications used in PsA within the context of glucose disorders, shedding light on their potential benefits.

## 2. Methods

A comprehensive systematic search was conducted in PubMed/MedLine and ScienceDirect, enclosing articles in English and Polish from December 2021 to July 2022. No specific year restrictions were applied for articles related to the pathomechanism of glucose disorders, psoriasis, and epidemiology to gather a broad database. However, for treatment recommendations, the search was limited to articles published between 2010 to 2022 to ensure the most recent findings. A search was performed using relevant keywords or title headings using the keywords as single terms or in combination: “diabetes mellitus”, “psoriasis”, “psoriatic arthritis”, “glucose disorders”, “hyperglycemia”, “obesity”, “epidemiology”, “pathomechanism”, “prevalence”, “insulin resistance”, “adipokines”, “chronic inflammation”, “treatment”, “guidelines”, “methotrexate”, “DMARDs”, “TNF-α.” In cases where more general keywords were used, factors such as the number of citations or publication date were applied to find the best matches. The screening process involved reviewing titles and abstracts followed by an examination of full articles by each mentioned author. Various article types, including original studies, reviews, observational studies, and clinical trials, were considered if they met the inclusion criteria. Articles were excluded if they were duplicated or addressed other disorders unrelated to PsA. The selected studies were individually reviewed by the first and second authors to ensure accuracy and to compare findings.

## 3. Epidemiology of Glucose Impairment in Patients with Psoriatic Arthritis

The prevalence of PsA exhibits geographical variations, with higher rates observed in the US and Europe (0.06–0.25% and 0.05–0.21%, respectively), while the lower prevalence is in South America and Asia, with no display of gender predominance [8]. The risk of PsA development increases when it coexists with psoriasis, with a prevalence ranging from 8% to 36.4% [7]. Dermatologic manifestations, such as nail dystrophy and evidence of scalp and perianal lesions, further contribute to the likelihood of developing PsA [9,10]. Moreover, obesity in early adulthood is associated with an elevated risk of developing PsA, presumably attributed to the synthesis of pro-inflammatory cytokines by adipose tissue [11]. 

Compared to the general population, with a DMt2 prevalence ranging from 2.4% to 14.8%, the prevalence of developing DMt2 in patients with PsA is notably higher, ranging from 6.1 to 20.2% [7]. PsA is strongly correlated with obesity and metabolic syndrome (MetS), whose prevalence is drastically higher, nearly 40%, compared to other rheumatic diseases. In comparison, the risk of metabolic disorders in rheumatoid arthritis accounts for 20%, while in ankylosing spondylitis for 11% [12,13]. Moreover, patients with late-onset psoriasis (occurring after age 40) and females face a greater risk of developing DMt2 [10,14]. Due to this disparity, patients with PsA should be regularly monitored and assessed for metabolic risk factors, particularly pertaining to glucose levels.

## 4. Mechanisms of Increased Glucose Levels in PsA

**Figure 1:**
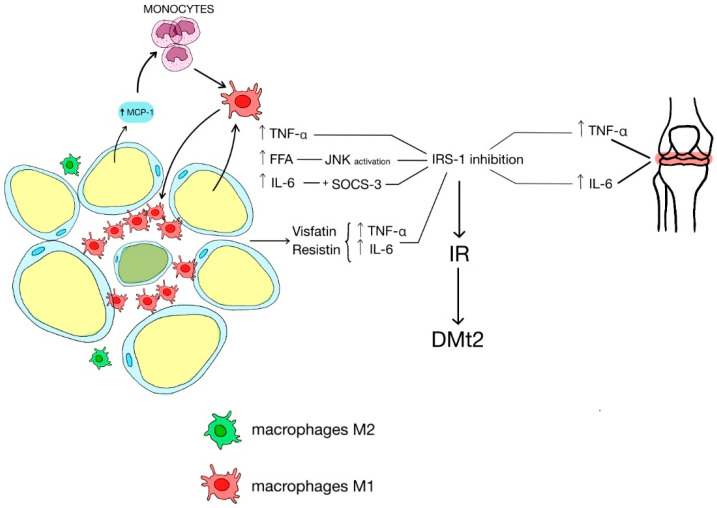
Figure 1 illustrates metabolic pathways involved in obesity and inflammation in PsA; In obesity, adipocytes undergo hypertrophy and produce chemokine MCP-1, which recruits monocytes locally. This results in the infiltration of M1 macrophages and the formation of crown-like structures. Cumulated M1 macrophages accumulate and produce MCP-1 along with other pro-inflammatory molecules such as IL-6, TNF-α, and FFA, further stimulating the immune response in macrophages and adipocytes. IL-6 and TNF-α are particularly associated with an inflammatory state seen in PsA. Additionally, visfatin and resistin play a role in IR by producing TNF-α and IL-6. These cytokines, along with the elevated levels of FFA, can inhibit IRS-1, resulting in progressive insulin resistance and DMt2.

Glucose disorders in patients with PsA are characterized by a multidimensional background. The pathomechanism involves the synthesis of inflammatory factors’ synthesis, which express a crucial role in inducing insulin resistance (IR) and DMt2 (Figure 1).

Insulin resistance (IR) is a significant component of DMt2 and is seen in almost 90% of diabetic patients. Its prevalence is higher in patients with PsA compared to other rheumatic diseases [15,16]. This condition is observed by increased hepatic glucose production and impaired glucose transport to the cells due to the downregulation of the GLUT-4 protein, leading to improper glucose uptake by the muscles. GLUT-4 protein is crucial for glucose regulation as it facilitates the transport of glucose to myocytes and adipocytes from the bloodstream. The complex process of docking GLUT-4 protein to the cell membrane relies on insulin activity [15,17]. Targeted cells contain insulin receptors composed of two alpha and two beta subunits. When insulin binds to the alpha subunit, it causes autophosphorylation of the beta subunits. As a result, phospho-tyrosine acts as a secondary messenger, catalyzing the phosphorylation of specific insulin receptor substrates (IRS). Enzyme PI-3K is activated by binding to IRS-1 through the Src homology 2 (SH2) domain, leading to the phosphorylation of phosphatidylinositol (4,5)-bisphosphate (PIP2), converting it into phosphatidylinositol (3,4,5)-trisphosphate (PIP3). PIP3, in turn, activates phosphoinositide-dependent kinase 1 (PDK-1), further activating protein kinase B (PKB) and protein kinase Cα (PKCα). These kinases, when activated, facilitate clathrin-aided movement of the GLUT-4 from GLUT-4-containing vesicles, promoting its translocation and docking into the cell membrane [18,19].

Some of the abbreviations used throughout this paper include: (CLS)—crown-like structures, (MCP-1)—monocyte chemoattractant protein-1, (TNF-α)—tumor necrosis factor Î±, (FFA)—free fatty acids, (JNK)—c-Jun N-terminal kinase, (IL-6)—interleukin 6, (SOCS-3)—suppressor of cytokine signaling protein 3, (IRS-1)—insulin receptor substrate 1, (IR)—insulin resistance, (DMt2)—diabetes mellitus type 2.

**Figure 2:**
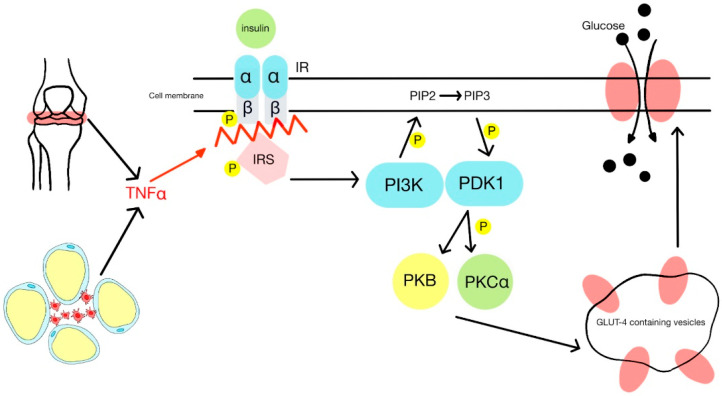
The mechanism of downregulation of GLUT-4 protein by the TNF-α activity. Insulin receptors consisting of two alpha and two beta subunits can be autophosphorylated when bound with insulin. That process results in the phosphorylation of IRS and can be disrupted by TNF-α synthesized during chronic inflammation in adipose tissue or arthritis. Without autophosphorylation, the PI3K cannot transform PIP2 to PIP3, and PDK-1, PKB, and PKCa are not activated. Due to that, GLUT-4 cannot be translocated and properly docked into the cell membrane, impairing the glucose intake to the cell. Abbreviations used in Figure 2 include: (TNF-α)—tumor necrosis factor-a, (IRS)—insulin specific substrates, (PI3K)—Phosphoinositide 3-kinases, (PIP2)—phosphatidylinositol 4,5-bisphosphate, (PIP3)—phosphatidylinositol (3,4,5)-trisphosphate, (PDK-1)—Phosphoinositide-dependent kinase-1, (PKB)—protein kinase B, (PKCα)—protein kinase Cα.

In PsA, elevated cytokine, particularly TNF-α, interferes with the autophosphorylation of insulin receptors resulting in impaired docking of the GLUT-4 transporter and increased IR [7], as shown in Figure 2. This pro-inflammatory cytokine, TNF-α, plays a critical role in the pathogenesis of IR by phosphorylating serine residues in IRS-1, thereby down-regulating the activity of PI-3 kinase and decreasing the expression of GLUT-4 [5,6]. Furthermore, TNF-α also activates the 11-beta-hydroxysteroid dehydrogenase 1, which converts inactive cortisone to its active form. Cortisol is known for its ability to lower peripheral insulin sensitivity by limiting the translocation of glucose transporters to the cell membrane [7].

Elevated levels of IL-6, a cytokine released by adipocytes, particularly visceral fat tissue, have been identified as another critical component associated with IR development [20]. IL-6 interferes with insulin signaling and cooperates with suppressors of cytokine signaling proteins (SOCS). Among these proteins is isotype SOCS-3, which acts as a negative regulator of insulin signaling through direct interaction with the insulin receptor. Moreover, IL-6 is involved in the induction of SOCS-3 expression, leading to the down-regulation of tyrosine phosphorylation of IRS, causing impaired insulin signaling [21,22].

The dysregulation of insulin signaling caused by IL-6 and SOCS-3 disruption contributes to the development of IR in patients with PsA. 

Low-grade chronic inflammation in adipocyte tissue increases the risk of metabolic disorders and influences the course of PsA. Obesity is associated with adipocyte hypertrophy and the synthesis of crown-like structures (CLS), which are clusters of dead fat cells surrounded by macrophages causing local inflammation [4]. This local inflammatory state in adipose tissue stimulates various groups of macrophages [23]. Among these macrophages, the M2 subtype is primarily involved in anti-inflammatory responses. In non-obese patients, M2 macrophages infiltrate fat tissue and synthesize physiological cytokines such as IL-4, IL-10, and IL-13, contributing to maintaining a balanced and healthy adipose tissue environment [20]. Unfortunately, in obesity, M1 macrophages dominate and produce IL-1, IL-6, TNF-α, and MCP-1, leading to monocyte infiltration of the fat tissue. Additionally, M1 macrophages and adipocytes contain TLR-4 receptors that can be activated by increased free fatty acids (FFA) produced by hypertrophic fat tissue. As a result, FFA triggers an inflammatory process creating a self-sustaining circle of self-driving inflammatory responses related to IR, a characteristic feature of obese patients with PsA [24].

In obesity-induced IR, there is increased activity of specific kinases. One key player in this process is the elevated activity of JNK, which is derived from the mitogen-activated protein (MAP) family. JNK can be activated by various factors, including cytokines, oxidative stress, radiation, and elevated FFA levels. Interestingly, once activated, JNK can phosphorylate serine in IRS-1; thus, it directly inhibits the insulin signaling pathway, similar to the effect of TNF-α. Moreover, synovial biopsies obtained from the knee joints of PsA patients demonstrated elevated activation of JNK in the perivascular compartment and lining layer [25]. This finding suggests that increased JNK activity is associated with arthritis and obesity, which often coexists in PsA individuals [5,6,25,26,27].

## 5. Adipokines Influence on Glucose Disorders

An increased amount of adipose tissue leads to excessive secretion of adipocytokines, which causes low-grade inflammation [28,29]. Increased levels of TNF-α, adiponectin, resistin, visfatin, and interleukin-6 (IL-6) are also observed in patients with PsA [30,31,32].

Visfatin, a pro-inflammatory adipokine released mainly by visceral tissue, influences the synthesis of TNF-α, IL-1B, IL-6, and chemokines interfering with glucose control [33]. Numerous studies confirmed that increased visfatin levels in patients with DMt2 are linked to the deterioration of pancreas B-cell function [34,35,36,37]. However, Dikbas et al. found no correlation between increased levels of adipokines, including visfatin, and PsA activity assessed by the Disease Activity in PSoriatic Arthritis (DAPSA) score [38]. The DAPSA score is used to evaluate disease severity by considering the number of swollen and tender joints, CRP levels, and the patient’s assessment of the pain and activity related to the disorder [39].

Resistin, another pro-inflammatory cytokine, is related to glucose disorders in PsA patients. This molecule links obesity and inflammation by inducing an immune response [40,41]. Interestingly, synovial fluid leukocytes can also produce IL-6 and TNF-α, stimulating resistin levels. Moreover, resistin accumulates in synovial fluid in patients with inflammatory arthritis as compared to degenerative or traumatic joint diseases. Animal studies have shown that injecting resistin at concentrations similar to those found in inflammatory arthritis into the joint space of mice induced leukocyte infiltration and synovial hyperplasia. However, the circulating resistin levels are usually low, suggesting local accumulation of this adipocytokine at the site of inflammation [42].

## 6. Psoriatic Skin Changes and Hyperglycemia

Psoriasis is an autoinflammatory papulosquamous skin condition characterized by reddish plaques with silvery-white scales, predominantly observed on the knees, elbows, scalp, and lumbosacral regions [43]. Disorders of the immune response lead to hyperproliferation and impaired differentiation of the keratinocytes. Multiple factors contribute to the complex pathomechanism of psoriasis, particularly the development of plaque. The immune response plays a critical role in the TNF-α/IL-23/Th17/IL-17 axis, where there are interactions between different immune cells, producing these pro-inflammatory molecules. Dysregulation of this axis is what contributes to chronic inflammation and keratinocyte proliferation observed in psoriatic plaques. In addition to immune-related factors, genetic, epigenetic, immunological, microbiome, and environmental factors also contribute to the development and severity of psoriasis, leading to clinical manifestations and progression of the disease [44].

Psoriasis, in addition to PsA, is an independent risk factor for developing DMt2 [45,46]. The mechanism of hyperglycemia in psoriasis is linked to increased serum levels of IL-17 and TNF-α. Psoriatic patients have significantly higher serum concentrations of IL-17 and TNF-α compared to the general population [47,48,49]. Studies on the imiquimod-induced psoriasis mice models (IMQ) showed increased fasting glucose levels and impaired insulin secretion compared to control healthy animals [50]. Additionally, pancreatic islets express receptors for specific cytokines, including IL-17 [49]. Therefore, administration of an antibody against IL-17A, commonly used in psoriasis treatment, decreases fasting glucose levels and restores islet functions in IMQ mice.

Clinical studies have demonstrated a correlation between the severity of psoriatic skin changes, as measured by psoriasis area and severity index (PASI), and higher glucose and hemoglobin A1c levels (HbA1c). This association suggests a significant link between psoriatic skin changes and glucose metabolism. A strong correlation is observed between HbA1c levels and the erythema component of PASI, emphasizing the inflammatory nature of the disease [50]. 

## 7. Effectiveness of Reducing Hyperglycemia during the Standard Treatment of PsA

The primary objective of treating PsA is not only managing the symptoms but also controlling the comorbidities. With an increasing number of new therapies available for patients with PsA, physicians now have more alternatives to choose from based on specific joint involvement and metabolic disorders of the patient. Treatment options include targeted therapies such as disease-modifying antirheumatic drugs (DMARDs) as well as symptomatic management with glucocorticosteroids (GCs) or non-steroidal anti-inflammatory drugs (NSAIDs). GCs recommended at the lowest doses by the European League Against Rheumatism (EULAR) are still commonly used during flare-ups of PsA, mainly as an adjunctive therapy. However, it is well known that the connection between GCs contributes to the development of DMt2; therefore, GCs’ use should be cautious and limited to short periods. Topical administration is preferred, while oral use is reserved for severe cases of PsA [51,52].

Methotrexate (MTX) is a frequently prescribed first-line conventional DMARD therapy in PsA and is also used as a systemic treatment for psoriasis [52]. MTX has demonstrated efficacy not only in PsA treatment but also in reducing HbA1c levels in non-diabetic patients. However, it does not improve insulin resistance, as measured by the Homeostatic Model Assessment of Insulin Resistance (HOMA-IR). There are conflicting findings concerning the role of MTX in managing glucose metabolism. While some studies report a significant decrease in HbA1c levels in patients treated with MTX, indicating an improvement in glucose metabolism, other studies, such as the one conducted by Dehpouri et al., showed no significant decrease in HbA1c levels in patients treated with MTX. Despite this conflicting information, MTX is considered a safe therapeutic option for psoriatic and PsA patients with metabolic comorbidities and remains an important component of the treatment strategy due to its effectiveness in managing PsA symptoms [53,54]. 

NSAIDs are commonly prescribed as the preferred treatment for the axial presentation of PsA and are associated with potentially beneficial effects on glucose metabolism. Studies indicate that NSAIDs can enhance glucose-induced insulin release and improve glucose tolerance in patients with type 2 diabetes [55]. However, it is important to note that NSAIDs may not be suitable for all patients due to serious side effects that partially correspond with comorbidities often seen in diabetes (e.g., hypertension). Moreover, there is a correlation between NSAIDs’ use and the development of chronic kidney failure among patients with DMt2 [56].

Effective therapeutic options for patients with PsA include the use of biological and targeted DMARD therapies. Recent studies show the effectiveness of various agents that target specific molecules and pathways involved in the pathogenesis of PsA. These targeted therapies include TNF-α, IL-17, IL-12/23, JAK/STAT inhibitors, and CTLA-4 immunoglobulin. These treatments aim to reduce disease activity, manage symptoms, and improve overall outcomes for patients with PsA.

TNF-α inhibitors are widely used in PsA treatment; however, similarly to MTX, their influence on glucose metabolism remains uncertain. A study conducted by Mantravadi et al. comparing MTX and TNF-α inhibitors in diabetic patients with PsA demonstrates similar reductions in HbA1c levels with both treatment methods [57]. Unfortunately, there are no studies analyzing selected anti-TNF-α influence on glucose disorders in PsA. Moreover, investigations on other rheumatic disease patients with DMt2 have indicated an improved insulin sensitivity measured by HOMA-IR in non-diabetic patients and decreased HbA1c and fasting glucose levels in diabetic patients with rheumatoid arthritis following treatment with anti-TNF-α drugs including infliximab, adalimumab, or etanercept [58,59,60]. It is important to note that conflicting results have emerged. For instance, a randomized study involving 1200 psoriatic patients taking adalimumab (TNF-α inhibitor) showed no effect on fasting glucose levels [61]. Da Silva et al. obtained a similar result, which revealed that treatment with anti-TNF-α had no impact on glucose metabolism in PsA patients without metabolic disorders [62].

In addition to the previously mentioned TNF-α and MTX, targeted DMARDs, such as JAK1/2 inhibitors, have emerged as potential treatment options for PsA. Medications, including baricitinib, upadacitinib, and tofacitinib, have been shown to successfully restore insulin function by lowering fasting glucose levels in mouse models; this effect is attributed to their ability to reduce low-grade inflammation [44]. However, it is worth noting that increased levels of IL-17 in PsA are linked to the development of IR. Nevertheless, studies show that both ixekizumab and secukinumab (IL-17A antibody) do not demonstrate improvements in glucose metabolism in patients with PsA or psoriasis [47,48,63,64,65,66]. Therefore, managing DMt2 in PsA patients remains a challenge and requires a more comprehensive approach that includes the treatment of diabetes itself and active management of inflammatory arthritis.

Given that many PsA patients have metabolic syndrome, obesity should be effectively treated together with PsA. This indication is significant because obesity negatively affects the pharmacokinetics of anti-TNF-α inhibitors, decreasing their effectiveness. Therefore, addressing obesity through weight loss interventions is of utmost importance. Studies show that a reduction in body mass by just 5% significantly improves the chances of achieving minimal disease activity, thereby offering a dual positive effect of enhancing treatment efficacy and reducing the severity of the PsA symptoms themselves [67,68].

Due to the possible risk of developing DMt2 when treating psoriasis and PsA, caution should be exercised when administering certain drugs, particularly in obese patients [10,69,70]. One such drug is oral isotretinoin, which might increase IR and raise the risk of developing DMt2 [71]. Fortunately, retinoids, including isotretinoin, are not typically the first choice in patients with psoriasis associated with metabolic disorders. 

In summary, adequate therapy of PsA and glucose disorders should consider the activity of arthritis comorbidities and patient preferences. Understanding the mechanism of action of the drugs used in PsA management and their influence on metabolic pathways allows for informed decision-making when choosing the best therapy, particularly for patients with concurrent glucose disorders. 

## 8. Diabetes Mellitus Type 2 Treatment in PsA Patients

Treatment of DMt2 continues to be a fascinating topic due to the increasing number of newly diagnosed cases. As a result of the numerous additional conditions that most patients with PsA experience, it is important to personalize management. Comorbidities such as obesity, kidney and heart failure, age, and general condition should be carefully evaluated before starting a new therapy [72].

The primary goal of DMt2 management is to increase insulin availability and achieve normoglycemia by improving insulin sensitivity or by direct insulin administration. Currently, there are two primary treatment approaches, including oral medications and insulin injections [71,72,73,74,75,76,77,78,79,80]. However, pharmacotherapy should always be followed by a lifestyle modification consisting of individualized nutrition therapy and exercises to normalize body mass. This holistic treatment can potentially create improved outcomes not only in DMt2 but also for PsA therapy [67,73,74].

Metformin is a first-line therapy drug administered to newly diagnosed patients with DMt2. It is contraindicated in renal insufficiency and significant GI disorders. Interestingly, metformin stimulates AMP-activated protein kinase (AMPK), which shares a similar mechanism of action as MTX. This led to the investigation of metformin as an additional therapy to MTX in PsA associated with DMt2, creating positive treatment outcomes. Recent studies demonstrated that the patients treated with metformin combined with MTX showed lower levels of pro-inflammatory cytokines. They also achieved better results in the American College of Rheumatology Criteria (ACR20) and Health Assessment Questionnaire-Disability Index (HAQ-DI) compared to the control group. Moreover, improvement in BMI and HOMA was observed in the intervention group. Apart from its positive effect on the PsA treatment, metformin also reduces the risk of macrovascular disease and does not cause a rise in body mass [75,76].

Glucagon-like Peptide-1 (GLP-1) drugs are a favorable choice medication option for patients with cardiovascular risks as they can potentially reduce major adverse cardiovascular events and diabetic kidney disease [77,78,79]. Keser M et al. found no correlation between PsA inflammation and GLP-1 treatment, suggesting that these drugs are considered to be safe [80]. 

A study performed by Faurschou et al. involving 20 obese patients with psoriasis has shown no significant improvement in PASI score after an 8-week-long treatment with liraglutide [81]. In contrast, another study revealed improvements in PASI scores during liraglutide therapy [82]. Similarly, spectacular results were obtained with semaglutide showing a 92% decrease in PASI compared with the baseline. Additionally, patients exhibited improvement in BMI and glycated hemoglobin (HbA1c) levels and a great reduction in dermatology life quality index (DLQI) from 26.0 (indicating an extremely negative effect on the patient’s life) to 0, demonstrating no impact of plaque psoriasis on the patient’s life [83].

The current limiting factors in establishing the direct influence of GLP-1 analogs on patients with PsA are related to the small sample sizes. Therefore, more research is needed in order to estimate their effect on psoriasis precisely. Nevertheless, the positive impact of GLP-1 drugs on weight loss and appetite control is confirmed, enabling obesity treatment to be more effective. Due to the adverse effects of obesity on psoriasis and PsA, these drugs could indirectly improve both conditions [84]. Thus, GLP-1 analogs may be a suitable option for obese patients with PsA due to their ability to diminish low-grade inflammation caused by excessive fat tissue. 

The use of sodium-glucose cotransporter-2 (SGLT2) inhibitors, while offering several positive outcomes such as decreased body mass, improved blood pressure control, reduced cardiovascular mortality, and protection against nephropathy, should be approached with caution as side effects of urinary tract infections and vulvovaginal candidiasis can also be observed. It is important to consider that exposure to infection might raise the risk of PsA flare. Thus, careful analysis is required before the implementation of SGLT2 inhibitors in this specific group of patients [72,85].

Similarly, SGLT2 or alpha-glucosidase inhibitors (AGIs) should be used with caution due to their ability to inhibit carbohydrate digestion and lower blood glucose levels. Their use, however, may increase the risk of psoriatic disease in patients with DMt2. Thus, the SGLT2 inhibitors should be administered thoughtfully, considering the impact on PsA patients with skin involvement. Further studies are needed to provide more comprehensive insights and better approaches [86].

Pioglitazone is an anti-diabetic medication that selectively stimulates the nuclear receptor peroxisome proliferator-activated receptor gamma (PPAR-γ). Its activation reduces IR by increasing glucose uptake and utilization and decreasing gluconeogenesis. Furthermore, PPAR-γ agonists decrease TNF-α levels and angiogenesis. During a 12-week study, Bongartz T. et al. analyzed the effect of pioglitazone in a group of patients with PsA and achieved promising results. The results showed a mean percentage reduction of 38% in PASI, and five patients attained the ACR20 response, indicating an improvement in PsA symptoms. While pioglitazone shows potential benefits, we must consider common side effects, such as fluid retention, that can worsen heart failure or the risk of anemia [87,88]. 

There may come a time when patients with DMt2 require insulin injections to achieve the expected glucose level. When choosing the best therapy option for patients, the treatment effectiveness and the influence on the best quality of life should be considered. Currently, two main types of insulin delivery modalities are utilized: continuous subcutaneous insulin infusion (CSII) and multiple daily injections (MDI) [89]. 

Achieving normoglycemia is crucial to prevent diabetic complications in patients and avoid the pro-inflammatory synthesis of IL-1β, IL-6, and TNF-α in a hyperglycemic state. Chronic inflammation is a pathological condition also occurring in PsA and obesity. Potentiated stimulation of cytokines might exacerbate PsA activity and result in disease flare-ups. IL-1β, IL-6, and TNF-α interact with glucose metabolism, worsening the patient’s glycemic state and escalating inflammation, creating an unending circle that can be halted by proper normoglycemic therapy [1,4,90,91].

## 9. Non-Pharmacological Recommendations for Hyperglycemia and DMt2 Treatment in PsA

When chronic impairment of glucose metabolism is not detected or appropriately treated, it causes an increased risk of systemic complications, like kidney failure, macro-and microvascular complications, and neuropathy [92,93]. However, the European (EULAR) and American (ACR) recommendations do not suggest guidelines for treating hyperglycemia in PsA. Nevertheless, other factors that increase the risk of diabetes development (obesity, severe psoriatic skin changes) should be effectively managed during hyperglycemia treatment (Table 1). Consequently, decreased inflammatory activity and glucose disorders can potentiate the probability of more effective treatment of PsA.

A balanced low-energy diet is considered an essential treatment for overweight or obesity. The Mediterranean diet has shown benefits in the context of PsA and DMt2, respectively, with significant reductions in DAPSA score and HbA1c levels compared to control groups not undergoing dietary modification [94,95]. Effective management of PsA and DMT2 necessitates a combination of a healthy diet and regular physical activity. Kessler et al. demonstrate that physical activity reduces pain, diminishes disease activity in PsA patients, and enhances their quality of life [73]. Similarly, in DMt2, physical activity influences insulin sensitivity, glycemic control, and body fat composition [96].

Modifying patient behavior during PsA treatment should encompass smoking cessation. Numerous studies highlight the impact of smoking on glucose metabolism and cardiovascular risk. For instance, a comprehensive cross-section study including 6089 participants revealed a clear correlation between smoking and elevated HbA1c levels [97]. Despite its association with weight loss, smoking increases the risk of developing abdominal obesity characterized by visceral adipose tissue deposition, which is more concerning than gynoidal fat distribution. Visceral adiposity triggers the release of inflammatory cytokines and is closely linked to a high risk of metabolic disorders in PsA patients [98].

**Table 1:** Treatment characteristics in patients with PsA and DMt2.

Treatment Options in Patients with PsA and DMt2			
Treatment	Specific Recommendation	Characteristics	Reference
non-pharmacological	Body mass reduction	↓ of inflammation ↓ of developing DMt2 or PsA ↑ response to treatment with anti-TNF-α inhibitors	[12,30,67,68]
	Well-balanced diet	↓ DAPSA score and ↓HbA1c by the Mediterranean diet	[90,91]
	Physical activity	↓ pain and disease severity ↑ life quality in PsA patients better DMt2 management	[73,92]
	Cessation of smoking	↓ HbA1c levels ↓ abdominal visceral fat deposition	[94]
	Treatment of chronic inflammation sources	↓ PsA symptoms ↑ IR and fasting glucose levels	[96,97]
	Practice stress-relieving techniques	↓ risk of DMt2 ↓ flare-up in PsA	[93,98]
pharmacological	Successful treatment of psoriasis	↓ psoriasis severity => ↓risks of developing DMt2 and PsA	[10,70]
	Avoiding GCS and isotretinoin in psoriasis treatment	↓ chances of developing IR and DMt2	[51,71]
	MTX administration	↓ in HbA1c levels in non-diabetic patients no improvement in HOMA-IR no improvement in HbA1c	[52,53,54]
	NSAIDs	↑ glucose tolerance and insulin release	[55]
	TNF-α	↑ IR measured by HOMA-IR and ↑ HbA1c and fasting glucose levels in non-diabetic patients with RA no effect on fasting glucose levels in psoriatic patients no impact on glucose metabolism in PsA	[58,59,60,61,62]
	JAK1/2 inhibitors	↓ fasting glucose levels on mice models	[63]
	IL-17 antibody	no effect on glucose metabolism in psoriatic/PsA patients	[47,48,64,65,66]
Combined testament	MTX and metformin	↓ levels of pro-inflammatory cytokines better results in ACR20 and HAQ-DI	[75,76]

Diabetes mellitus type 2 (DMt2), psoriatic arthritis (PsA), glucocorticosteroids (GCS), non-steroidal anti-inflammatory drugs (NSAIDs), Disease Activity in PSoriatic Arthritis (DAPSA), Homeostatic Model Assessment of Insulin Resistance (HOMA-IR), methotrexate (MTX), insulin resistance (IR), hemoglobin A1c (HbA1c). ↑: increase. ↓: decrease.

Moreover, the pro-inflammatory state typical of obesity heightens the susceptibility to infections, a risk compounded by the use of immunosuppressive drugs used in PsA. Consequently, exposure to infections in the presence of chronic inflammation elevates the severity of PsA symptoms. Patients with tonsillitis or periodontal issues should consider interventions such as tonsillectomy or improve their oral health practices [99]. While the precise mechanism underlying the impact of pathogens on PsA activity remains incompletely understood, evidence is not fully known. However, it was proved that microorganisms exacerbate autoimmune disorders by activating immune cells, disrupting gene transcription and translation, and altering human cell metabolism [100]. 

Notably, Vasey et al. found that PsA patients exhibit higher serum levels of streptococcal exotoxins, such as anti-deoxyribonuclease-B, compared to patients with psoriasis or rheumatoid arthritis [101]. Periodontal conditions, common in PsA, contribute to increasing insulin resistance, leading to elevated blood glucose levels in individuals with diabetes. However, effective treatment of periodontal-associated conditions can reverse the hyperglycemic state. Furthermore, periodontosis exacerbates PsA symptoms, potentially compromising the efficacy of treatments [102]. The treatment of diabetic patients with diagnosed PsA is summarized in Table 2.

Stress is another critical factor that necessitates careful consideration in PsA treatment. Stress is a significant trigger factor for various autoimmune disorders, with a notable 31–88% of psoriasis patients attributing PsA flares to stress [103]. Similarly, stress serves as an underlying factor in the development of DMt2 [97]. Techniques including meditation, biofeedback, and hypnosis have successfully alleviated stressful conditions [103,104].

## 10. Conclusions

The challenges posed by glucose metabolism disturbances, such as IR or DMt2 in PsA, warrant further investigation to develop optimal strategies for affected patients. A comprehensive understanding of the molecular mechanisms involved is essential for the effective management of PsA in conjunction with glucose disorders. The presence of elevated adipokines and cytokines, coupled with a low-grade inflammatory state, disrupts glucose metabolism by impeding the proper function of GLUT-4, the formation of CLS, induction of SOCS-3 protein, and abnormal activation of JNK. These cascading effects ultimately result in metabolic disturbances. The role of adipokines (adiponectin, visfatin, and resistin) in developing autoimmune disorders and their potential impact on treatment’s efficacy, particularly in the context of PsA, remains uncertain and necessitates father studies. 

The recommended treatment options for PsA have non-conclusively demonstrated benefits for glucose metabolism disruptions, and ongoing investigations hold promise. MTX, TNF-α inhibitors, JAK1/2 inhibitors, and IL-17 antibodies yield inconsistent results regarding glycemic control in clinical trials though they appear safe for DMt2 patients. Given that GCS and isotretinoin can exacerbate PsA development, their usage for treating psoriatic skin changes should be minimized. Effective management of chronic inflammation such as tonsilitis or periodontosis issues is essential to mitigate the risk of worsening PsA and DMt2.

Preventive measures play a pivotal role in controlling and managing metabolic comorbidities in PsA. Apart from conventional medical therapy, patients should be advised to adopt lifestyle modifications, including smoking cessation, increased physical activity, and adherence to a balanced diet. These practices have been demonstrated to be highly beneficial in both preventing and controlling DMt2 and PsA. However, it is undeniable that PsA is associated with an increased risk of developing MetS. Concurrently, other conditions like hypertension, dyslipidemia, glucose intolerance, and increased cardiovascular risk should necessitate comprehensive multidisciplinary care for affected patients.

Attaining and maintaining normoglycemia treatment is a critical aspect of effective PsA treatment. It not only reduces complications associated with micro and macrovascular issues and nephropathy but also influences inflammation by decreasing cytokine levels. Elevated pro-inflammatory molecules have the potential to exacerbate the course of PsA. Certain anti-diabetic medications, including metformin or pioglitazone, exhibit positive effects on PsA, while others might have a neutral or negative impact on the disease. However, determining the appropriate treatment approach should always be tailored to the patient’s clinical status and coexisting comorbidities.

Identifying risk factors associated with metabolic disorders during PsA or psoriasis is pivotal in mitigating the risk of active arthritis and comorbidities linked to metabolic diseases. The management of PsA patients with associated clinical complications demands a holistic approach encompassing psychological support to modern immunosuppressive therapies. Nonetheless, future research is imperative to establish a well-validated strategy for managing glucose disorders in PsA patients. 

## Figures and Tables

**Table 2:** Anti-diabetic treatment of patients with PsA and DMt2.

Treatment of Diabetes Mellitus Type 2 in PsA Patients			
Type of Therapy	Drug/ Management	Characteristics of Management	Reference
non-pharmacological management	personalized management	individual modification of lifestyle factors, including dietary habits and physical activity	[67,73,74]
	metabolic parameters	balanced low-energy diet in obesity (e.g., the Mediterranean diet) => ↓DAPSA score and ↓HbA1c level physical activity => ↓pain, ↓PsA activity, ↑ QoL proinflammatory activity of adipose tissue => ↑ risk of infection => ↑ PsA severity smoking cessation => ↑ HbA1c and ↑ risk of visceral adiposity => ↑ metabolic disorders risk in PsA	[73,74,94,95,97,98]
	↓ stress level	meditation biofeedback hypnosis	[97,102,103,104]
pharmacological oral hypoglycemic therapy	metformin	first-line therapy in newly diagnosed DMt2 contraindicated in renal insufficiency or severe GI disorders stimulates AMPK—has a similar mechanism of action as MTX combined treatment: metformin and MTX => ↓pro-inflammatory cytokines, ↓BMI, ↓HOMA, ↓risk of macrovascular disease	[71,72,73,74,75,76]
	GLP-1	↓ major adverse CVD events and diabetic kidney disease safe in patients with PsA	[77,78,79,82,83,84]
	SGLT2 inhibitors	positive effects: ↓body mass, ↓hypertension, ↓CVD mortality, ↓risk of nephropathy adverse effects: ↑ risk of infections (e.g., urinary tract infections, vulvovaginal candidiasis) => ↑ risk of PsA flare	[72,85]
	AGIs	prevent carbohydrates’ absorption ↓ blood glucose combined therapy: AGIs and metformin => ↑ risk of psoriasis	[86]
	pioglitazone	selectively stimulates PPAR-γ => ↓IR, ↓TNF-α levels, ↓angiogenesis, ↓PASI adverse effect: fluid retentions => ↑ risk of heart failure or anemia	[87,88]
	insulin therapy	CSII MDI	[89]
other therapies	heart and vessel diseases	dyslipidemia treatment effective management of CVD disorders	[16,36,64,68,72,77,78,92]
	infection	the pro-inflammatory activity of adipose tissue => ↑ infection risk => ↑ PsA severity microorganisms causing periodontosis and tonsillitis => stimulate immune cells, interfere with gene transcription and translation, alter metabolic pathways periodontosis in PsA: ➢ ↑ IR => chronic hyperglycemic state ➢ exacerbates PsA symptoms and ↓ treatment’s effectiveness	[99,100,101,102]

AGIs—alpha-glucosidase inhibitors; AMPK—activated protein kinase; CSII—continuous subcutaneous insulin infusion; CVD—cardiovascular; DAPSA—Disease Activity in PSoriatic Arthritis; GI—gastrointestinal disorders; GLP-1—glucagon-like Peptide-1; IR—insulin resistance; MDI—multiple daily injections; QoL—quality of life; PASI—psoriasis area and severity index; PPAR-γ—peroxisome proliferator-activated receptor gamma; SGLT2—sodium-glucose cotransporter-2. ↑: increase. ↓: decrease.

## Data Availability

Not applicable.
